# The hippocampal sparing subtype of Alzheimer’s disease assessed in neuropathology and in vivo tau positron emission tomography: a systematic review

**DOI:** 10.1186/s40478-022-01471-z

**Published:** 2022-11-14

**Authors:** Daniel Ferreira, Rosaleena Mohanty, Melissa E. Murray, Agneta Nordberg, Kejal Kantarci, Eric Westman

**Affiliations:** 1 Division of Clinical Geriatrics; Center for Alzheimer Research; Department of Neurobiology, Care Sciences and Society, Karolinska Institutet, Blickagången 16 (NEO building, floor 7th), 14152 Huddinge, Stockholm, Sweden; 2 Department of Radiology, Mayo Clinic, Rochester, MN USA; 3 Department of Neuroscience, Mayo Clinic, Florida, USA; 4 Theme Aging, Karolinska University Hospital, Huddinge, Sweden; 5 Department of Neuroimaging, Center for Neuroimaging Sciences, Institute of Psychiatry, Psychology and Neuroscience, King’s College London, London, UK

**Keywords:** Alzheimer’s disease, Subtypes, Heterogeneity, Neuropathology, Neurofibrillary tangle, Positron emission tomography, Hippocampal sparing, Systematic review

## Abstract

Neuropathology and neuroimaging studies have identified several subtypes of Alzheimer’s disease (AD): hippocampal sparing AD, typical AD, and limbic predominant AD. An unresolved question is whether hippocampal sparing AD cases can present with neurofibrillary tangles (NFT) in association cortices while completely sparing the hippocampus. To address that question, we conducted a systematic review and performed original analyses on tau positron emission tomography (PET) data. We searched EMBASE, PubMed, and Web of Science databases until October 2022. We also implemented several methods for AD subtyping on tau PET to identify hippocampal sparing AD cases. Our findings show that seven out of the eight reviewed neuropathologic studies included cases at Braak stages IV or higher and therefore, could not identify hippocampal sparing cases with NFT completely sparing the hippocampus. In contrast, tau PET did identify AD participants with tracer retention in the association cortex while completely sparing the hippocampus. We conclude that tau PET can identify hippocampal sparing AD cases with NFT completely sparing the hippocampus. Based on the accumulating data, we suggest two possible pathways of tau spread: (1) a canonical pathway with early involvement of transentorhinal cortex and subsequent involvement of limbic regions and association cortices, and (2) a less common pathway that affects association cortices with limbic involvement observed at end stages of the disease or not at all.

**Supplementary Information:**

The online version contains supplementary material available at 10.1186/s40478-022-01471-z.

## Introduction

**Fig. 1:**
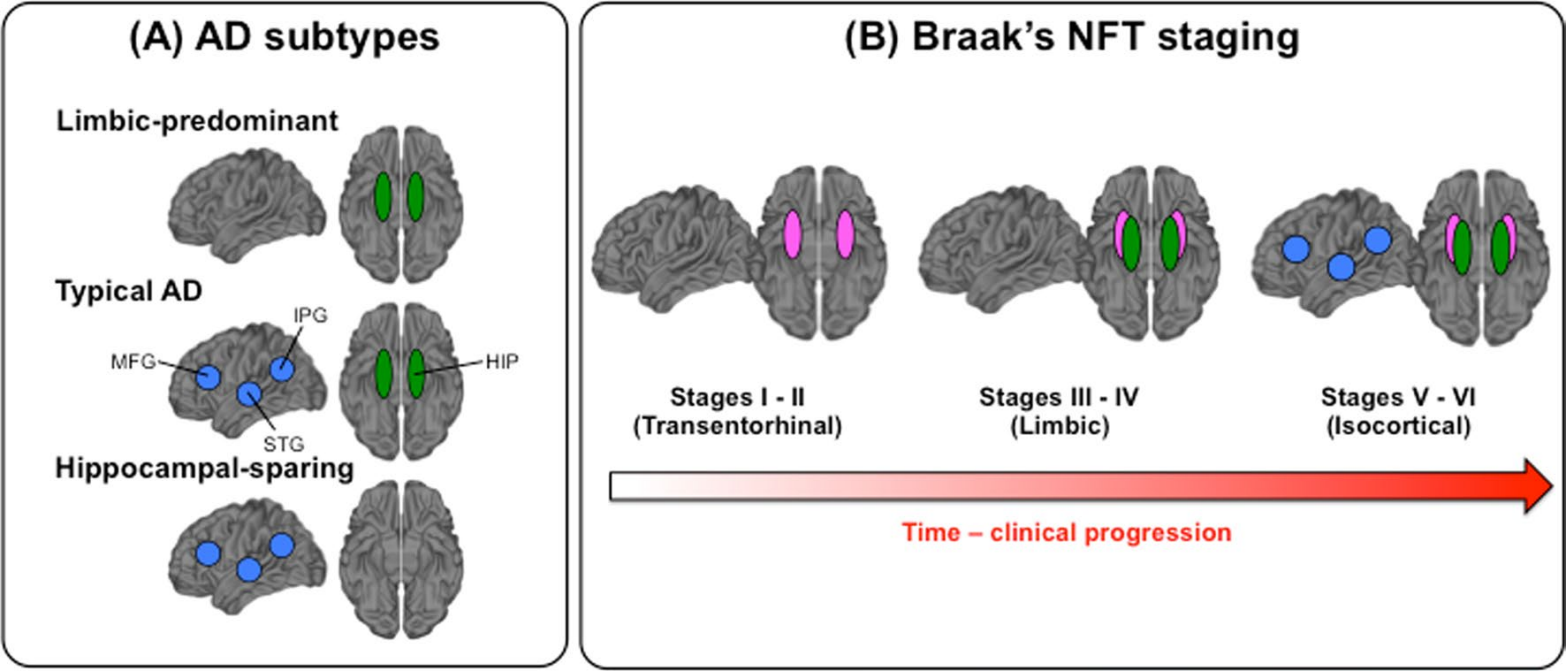
Schematic representation of AD subtypes and Braak’s NFT staging. **a** The panel shows the predominant location of tau neurofibrillary tangles (NFT) in hippocampus and association cortex, across subtypes, as described in Murray et al. [7]. In the figure, location of tau NFT is represented as green ellipsoids and blue circles. In green, hippocampus (HIP). In blue, association cortex, including medial frontal gyrus (MFG), superior temporal gyrus (STG), and inferior parietal gyrus (IPG). **b** Spreading of tau NFT as postulated by Braak and Braak [10], including stages from I to VI, over time. In green, hippocampus; in blue, association cortex; in pink, transentorhinal cortex (and Cornu Amonis 1 region of the hippocampus in Stage II). Abbreviations: AD = Alzheimer’s disease

The field of biological subtypes of Alzheimer’s disease (AD) has increasingly gained attention [1], envisioned to be a strong driver of precision medicine and future clinical trials [2]. Neuropathology and neuroimaging studies have consistently identified three subtypes based on the distribution of neurofibrillary tangle (NFT) pathology and patterns of brain atrophy [1, 3–7]: hippocampal sparing, limbic predominant, and typical AD (Fig. 1a). A fourth subtype known as minimal atrophy AD has also been identified in structural imaging studies [1, 3], and we recently described the minimal tau AD subtype on tau PET [8].

An important and still unresolved question is whether the hippocampal sparing subtype follows a different neuropathologic pathway than limbic predominant and typical AD subtypes. The hippocampal sparing subtype of AD is plausible in magnetic resonance imaging (MRI) studies because atrophy can occur in the association cortex while completely sparing the hippocampus (i.e., no atrophy in the hippocampus) [1, 3, 9]. However, the same pattern is questionable in neuropathology studies as NFT accumulating in the association cortices while completely sparing the hippocampus (i.e., no NFT in the hippocampus) would challenge the widely used model of neurofibrillary changes (NFT and neuropil threads) defined by Braak and Braak [10]. In that model, NFT in the hippocampus precede NFT accumulation in the association cortex. More specifically, Braak and Braak postulated that NFT accumulation typically starts in the transentorhinal cortex (Braak stage I), although a few isolated NFT may additionally occur in the entorhinal cortex, Cornu Ammonis 1 (CA1) region of the hippocampus, basal forebrain, and antero-dorsal nucleus of the thalamus [10]. Stage II includes modest numbers of NFT in the hippocampus (CA1). Stage III involves the entorhinal cortex, subiculum, other regions of the hippocampus (CA2-4), and the amygdala. Stage IV involves several subcortical gray matter structures such as the putamen and nucleus accumbens. Finally, although some NFT can reach the isocortex during stage III and IV, the association cortex is severely involved in stage V, and the primary sensory cortex in stage VI. These stages are summarized as transentorhinal (I–II), limbic (III–IV), and isocortical (V–VI) stages in Braak and Braak’s model (Fig. 1b). Hence, a strict definition of hippocampal sparing AD would imply that NFT reached the isocortex without involving the hippocampus.

The first report on hippocampal sparing AD was published in 2011 [7], including 889 cases with a neuropathologic diagnosis of AD. All the cases were at Braak stage [10] > IV, implying that they all had NFT both in the hippocampus and association cortex (as defined by the middle frontal, superior temporal, and inferior parietal cortices, Fig. 1a, b). In that study, hippocampal sparing AD was defined as cases with higher NFT counts in the association cortex compared to group average and lower hippocampal NFT counts compared to group average, with the ratio of hippocampal:cortical NFT counts being less than the 25th percentile to ensure classification of extreme phenotype [7] (a method later on referred to as the “Murray’s algorithm”). In the 2011 publication, hippocampal sparing relative to greater cortical involvement defines the phenotype [7].

**Fig. 2:**
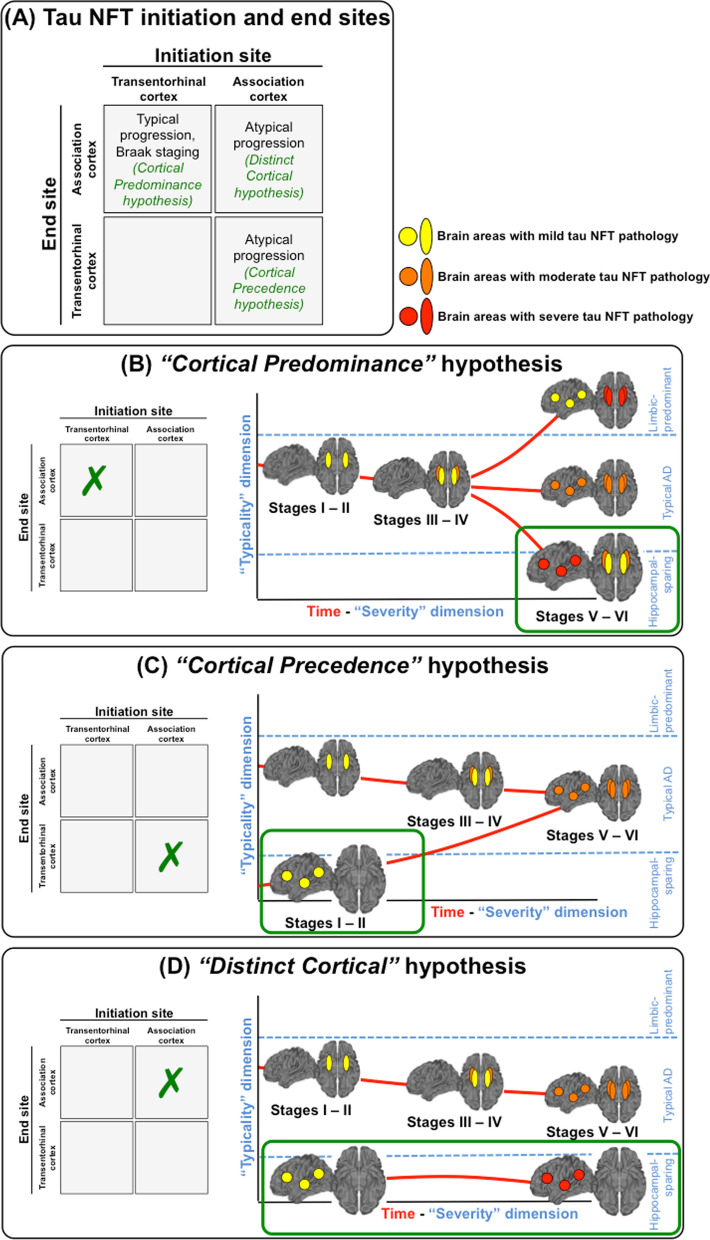
Three hypotheses of hippocampal sparing AD based on different initiation and end sites of tau pathology. Based on initiation and end sites of tau pathology (**a**), we hypothesized three possible scenarios: (**b**) NFT accumulation follows the stereotypical order defined by Braak and Braak [10], where NFT in the hippocampus always precede NFT in the association cortex. Hence, hippocampal sparing AD would only exist in Braak stage V and VI and reflects individuals with NFT predominantly in the association cortex. The colors represent mild (yellow), moderate (orange), and severe (red) degrees of tau pathology. Colors in panel b are just hypothetical examples to represent that in the hippocampal sparing subtype, tau pathology would reach a higher degree of pathology in the cortex (e.g. in red, severe degree), than in the hippocampus (e.g. in yellow, mild degree). However, other degrees of pathology are possible. For example, typical AD is defined by balanced degrees of pathology in cortex and hippocampus, so that the degrees of pathology can indeed be mild, moderate, or severe, while in our Fig. 2b we depicted them in orange for illustration purposes); (**c**) NFT accumulation in the association cortex precedes NFT accumulation in the hippocampus, occurring before Braak stage II, but all subtypes converge at Braak stage V/VI. Again, colors in panel c are just hypothetical examples and other degrees of pathology are also possible; and (**d**) NFT accumulates in the association cortex while completely sparing the hippocampus across the entire disease progression up to death. As for the other two hypotheses, colors in panel d are just hypothetical examples and other degrees of pathology are also possible. Only the “*distinct cortical”* hypothesis would support the existence of a strictly hippocampal sparing subtype of AD, since the “*cortical predominance*” and “*cortical precedence*” hypotheses imply the presence of tau NFT in the hippocampus. Panels b, c, and d show examples of the hippocampal sparing AD subtype, and the depicted colors are just examples, but other degrees of pathology are also possible as long as the level of pathology in cortex is higher than that in hippocampus (which defines this subtype). The figure has a focus on hippocampal sparing AD, and it does not provide examples for other subtypes such as limbic predominant AD or minimal tau AD, in all the panels. Abbreviations: AD = Alzheimer’s disease; NFT = neurofibrillary tangles

Given the emphasis on “relative sparing”, the goal of our current study was to investigate whether AD patients can have NFT in the association cortex while completely sparing the hippocampus, and to assess three possible neuropathologic pathways based on initiation and end sites of tau pathology (Fig. 2a): (i) NFT accumulation follows the stereotypical order defined by Braak and Braak [10], where NFT in the hippocampus always precede NFT in the association cortex. Hence, hippocampal sparing AD would only emerge at Braak stage V and VI and merely reflects cases with NFT counts predominantly in the association cortex (we will call this the “*cortical predominance*” hypothesis, Fig. 2b); (ii) NFT accumulation in the association cortex precedes NFT accumulation in the hippocampus, thus occurring any time before Braak stage II, but all subtypes converge at Braak stage V/VI (we will call this the “*cortical precedence*” hypothesis, Fig. 2c); and (iii) NFT accumulates in the association cortex while completely sparing the hippocampus across the entire disease progression up to death (we will call this the “*distinct cortical*” hypothesis, Fig. 2d). Only the “*distinct cortical”* hypothesis would fit with the strict definition of hippocampal sparing AD, which implies NFT completely sparing the hippocampus. In contrast, the “*cortical predominance*” and “*cortical precedence*” hypotheses imply the presence of NFT in the hippocampus. To address the goal of our study, we conducted a systematic review of the literature and further provide original data to gain novel insight.

## Materials and methods

### Systematic review

We capitalized on our previous systematic review [1], conducted on EMBASE, PubMed, and Web of Science databases as per the PRISMA statement, and performed an update of new publications up to October 2022. The search strategy combined the following medical subject heading (MeSH) and free-text terms (Additional file 1: Table S1): “Alzheimer”, “AD”, “subtype”, “heterogeneity”, “atrophy”, “patterns”, “subtypes”, “MRI”, “Magnetic Resonance”, “PET”, “postmortem”, “neurofibrillary tangle”, and “neuropathological”. Additional relevant publications were identified by scrutinizing references of the included papers.

Selection criteria for the current systematic review were: (i) case–control studies reporting data on NFT count or tau PET uptake in such a way that interpretations could be drawn with regards to potential hippocampal sparing AD cases (i.e., presence of NFT or abnormal tau PET values in the association cortex in conjunction with absence of NFT or normal tau PET values in hippocampus/entorhinal cortex); (ii) studies including participants in the AD continuum; (iii) articles published in English.

Study selection was performed by a single researcher (D.F.), involving a second researcher (E.W.) when needed. Several strategies were followed to reduce risks bias related to publication, data availability, and reviewer selection (Additional file 1: Table S2). Data extraction was performed by a single researcher (D.F.) including the fields listed in Additional file 1: Table S3. A studies’ methodological quality was assessed with the CASP checklist for case control studies.

### Original data

In addition to our systematic review, we re-analyzed the data published in three previous studies (Whitwell et al. [11], Charil et al. [6], and Young et al. [12]. We also produced brand new data using the Alzheimer’s Disease Neuroimaging Initiative (ADNI) cohort (please see below for a description of the ADNI cohort) [13]. This re-analysis was based on tau PET data. Due to the nature and idiosyncrasy of tau PET data, the ability to identify potential hippocampal sparing AD cases may be partially influenced by the cut points used to define abnormality in tau PET uptake. Hence, for this re-analysis, based on the figures provided in Whitwell et al. [11], Charil et al. [6], and Young et al. [12], we applied five complementary cut points for tau PET data in order to interpret abnormality using the tau PET tracer flortaucipir (18F-AV-1451), and re-classified participants into hippocampal sparing AD. The cut points are fully explained in Table 1. Briefly, the *‘accuracy-based cut point’* is increasingly used in the field but is conservative and is based on a meta-region of interest (ROI) that includes the entorhinal cortex and several other cortical areas (see Table 1 for a description of the meta-ROI) [14]. Hence, we also tested previously published less conservative cut points that are specific to hippocampus and entorhinal regions that we calculated using the publicly available ADNI data. These cut points are based on the + 1 standard deviation (SD) [15] of flortaucipir uptake in amyloid-negative cognitively unimpaired individuals; and the sensitivity (10th percentile from amyloid-positive cognitively impaired individuals) method [16]. We will refer to these cut points as *‘* + *1SD cut point’* and ‘*10% cut point*’. An advantage of less conservative cut points is that they might be more capable of capturing early cortical tau deposition [17]. Finally, we complemented our analyses by using two other popular cut points that are based on a data-driven method for staging individuals into transentorhinal, limbic, and isocortical Braak stages, as introduced by Schöll et al. [18] and Maass et al. [19](referred to as *‘Schöll cut point’* and *‘Maass cut point’*).

**Table 1:** Cut points for the determination of abnormal levels of flortaucipir uptake

Reference	Referred to as in our current study	Criterion	Region/s	Cut point	Data source
Jack et al. [14, 16]	*‘Accuracy-based cut point’*	Accuracy based on age-matched clinically normal versus amyloid-positive cognitively impaired individuals from the MCSA	Meta-ROI including entorhinal, amygdala, parahippocampal, fusiform, inferior temporal, and middle temporal ROIs	≥ 1.33	MCSA
Byun et al. [15]	*‘1SD cut point’*	Flortaucipir uptake + 1SD from amyloid-negative cognitively unimpaired individuals from ADNI	Hippocampus Entorhinal cortex	≥ 2.79 ≥ 3.73	ADNI
Jack et al. [16]	*‘10% cut point’*	Sensitivity (10th percentile flortaucipir uptake) based on amyloid-positive cognitively impaired study participants from ADNI	Hippocampus Entorhinal cortex	≥ 2.75 ≥ 3.83	ADNI
Schöll et al. [18]	*‘Schöll cut point’*	Conditional inference tree analysis to classify individuals into Braak stage I/II	Transentorhinal, hippocampus, and entorhinal cortex	≥ 1.40	BACS and UCSF-MAC
Maass et al. [19]	*‘Maass cut point’*	Conditional inference tree analysis to classify individuals into Braak stage I/II	Transentorhinal, hippocampus, and entorhinal cortex	≥ 1.13	BACS and UCSF-MAC

The methods for determination of abnormal levels of flortaucipir uptake are different (criterion column), and there is also additional methodological variation across the original studies. Partial volume correction was applied in our analysis of ADNI data and all the original studies except for the *‘accuracy-based cut point’*, although borderline voxels were discarded by the authors.^14,16^ The meta-ROI implemented in the *‘accuracy-based cut point’* includes amygdala, entorhinal cortex, fusiform, parahippocampal, and inferior temporal and middle temporal gyri. Hence, the meta-ROI does not include any of the regions used for subtyping in Murray et al. [7] i.e. superior temporal, middle frontal, and inferior parietal gyri. *MCSA* Mayo Clinic Study of Aging, *BACS *Berkeley Aging Cohort Study, *UCSF-MAC* University of California San Francisco—Memory and Aging Center, *ADNI *Alzheimer's Disease Neuroimaging Initiative, *ROI* region of interest, *SD* standard deviation, *pc* percentile

ADNI (http://adni.loni.usc.edu/) data retrieval, as well as tau PET collection and processing were previously described in detail in Mohanty et al. [8]. The goal of the ADNI (launched in 2003, PI: Michael W. Weiner) [13] is to measure the progression of prodromal AD and early AD using MRI, PET, and cerebrospinal fluid biomarkers, as well as clinical and neuropsychological assessments. Briefly, we selected participants from ADNI-2 and ADNI-3 who had a tau PET scan, including 84 participants (54 amyloid-beta positive prodromal AD participants, 30 amyloid-beta positive AD dementia participants) and 200 amyloid-beta negative cognitively unimpaired healthy controls. Amyloid status was determined through amyloid PET (florbetapir cut-off = 1.11 or florbetaben cut-off = 1.08) [8]. The ADNI study was performed in accordance with the ethical standards by the Declaration of Helsinki, and ethics committees at each participating center reviewed and approved data collection and study procedures. All participants / their legal guardians gave their informed consent prior to their inclusion in the ADNI study.

To answer the question of *“can AD cases have NFT in the association cortex while completely sparing the hippocampus (or entorhinal cortex)”*, we applied the following subtyping algorithms on the tau PET data from ADNI: Risacher et al. [20], Byun et al. [15], and Charil et al. [6]. These subtyping methods are thoroughly explained in the cited publications, and they were implemented as detailed in Mohanty et al. [8], so as to replicate the original method as closely as possible.

The accurate quantification of flortaucipir signal in the hippocampus is challenging, mostly due to off-target signal in choroid plexus [21]. Still, hippocampus is a key region for subtyping in many studies [6, 15, 20], as it is for Braak staging [10]. Hence, we approached this problem by applying partial volume correction in our analysis of ADNI data. The same was done in all the original studies we reviewed and re-analyzed, as well as for the generation of all cut points used (except for the ‘accuracy-based cut point’, where an alternative procedure was carried out). In addition, we applied subtyping using the entorhinal cortex instead of the hippocampus, as a control analysis. Entorhinal cortex was previously used for subtyping in tau PET studies [11, 22], thus providing a method for comparability with our current study. We used TheHiveDB for data management and processing [23].

### Statistical analyses

We report the number of cases that were classified as hippocampal sparing AD in the original studies and calculated respective percentages out of their total samples. Additionally, we used the ADNI cohort to calculate the *‘* + *1SD cut point’* and *‘10% cut point*’ for tau PET, as described in Table 1. The critical values for the *‘accuracy-based cut point’*, *‘Schöll cut point’*, and *‘Maass cut point’* were directly taken from the original publications [16, 18, 19] (see Table 1). Using these five alternative cut points, we examined the data presented in Whitwell et al. [11], Charil et al. [6], and Young et al. [12] in order to identify hippocampal sparing AD cases that had normal tau PET uptake values in the hippocampus or entorhinal cortex.

The ability to identify potential hippocampal sparing AD cases also depends on the subtyping algorithm used [8]. Hence, we additionally classified amyloid-positive prodromal AD and AD dementia participants from the ADNI cohort using three different subtyping algorithms [6, 15, 20] on the tau PET data, and used the five alternative cut points to identify hippocampal sparing AD participants who had normal tau PET uptake values in the hippocampus or entorhinal cortex. In all these analyses, we report the percentage and number of participants as the outcomes of interest.

## Results

### Systematic review

**Fig. 3:**
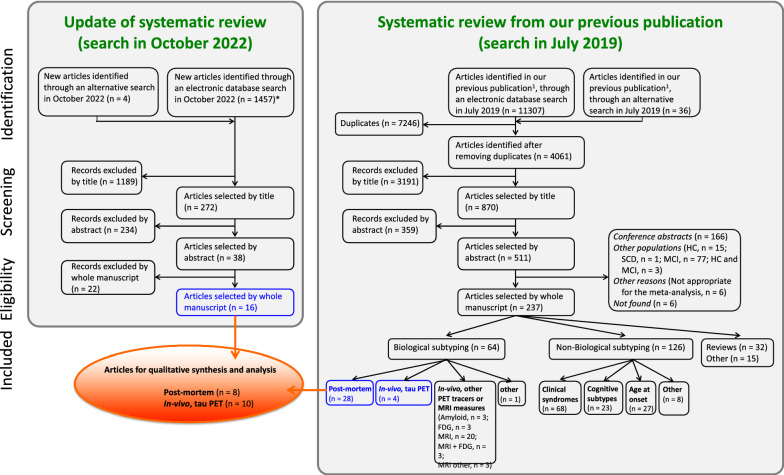
Study selection flowchart. By updating our search from July 2019 (right panel, n = 11,343 hits) through a new search in October 2022 (left panel, n = 1461), we identified 12,804 records. After removing duplicates and screening by title, abstracts, and full text, 48 records were selected (blue boxes). Of those, we further excluded 30 records because of the reasons listed in Additional file 1: Table S4. This gave a total of 18 studies for our qualitative synthesis and original analysis (orange ellipsoid). *The search in October 2022 used the same medical subject heading (MeSH) and free-text terms than in July 2019, but duplicates were removed automatically during the actual search strategy. *PET *positron emission tomography; *FDG *fluorodeoxyglucose; *MRI* magnetic resonance imaging; *HC* healthy control; *SCD* subjective cognitive decline, *MCI* mild cognitive impairment

Our search identified 12 804 records. After removing duplicates and screening by title, abstracts, and full text, 48 records were selected (Fig. 3, blue boxes). Of those, we excluded 30 records because of the reasons listed in Additional file 1: Table S4. This gave a total of 18 studies for our qualitative synthesis and original analysis (Fig. 3, orange ellipsoid). Table 2 shows the key characteristics of these studies. All the selected studies had an appropriate methodological quality according to the CASP checklist.

**Table 2:** Main characteristics of the studies included in the systematic review

Study	Cohort	Number of individuals with AD	Clinical phenotype	Age	Sex
*Postmortem studies*					
Murray et al. [7]	Mayo Clinic, Jacksonville, FL, US	889 (all at the AD dementia stage, with neuropathologic AD confirmation)	Typical and atypical AD	From 37 to 103 years (at death)	55% women
Hanna Al-Shaikh et al. [4]	Mayo Clinic, Jacksonville, FL, US	1 361 (all at the AD dementia stage, with neuropathologic AD confirmation)	Typical and atypical AD	From 54 to 104 years (at death)	54% women
Whitwell et al. [9]	Mayo Clinic, Rochester, MN, US	177 (all at the AD dementia stage, with neuropathologic AD confirmation)	Typical and atypical AD	From 65 to 95 years (at death)	60% women
Sahoo et al. [24]	Mayo Clinic, Rochester, MN, US	36 (all at the AD dementia stage, with neuropathologic AD confirmation)	Non-amnestic AD	From 52 to 98 years (at death)	39% women
Petersen et al. [25]	UCSF-MAC, CA, US	74 (all at the AD dementia stage, with neuropathologic AD confirmation)	Typical and atypical AD	71 years (average age at death for the whole cohort, N = 94)	43% women
Corder et al. [28]	Karolinska Institutet, Stockholm, Sweden	249 (all neurological patients, 62 were at the dementia stage—AD or other)	Heterogeneous clinical diagnoses	79 years (average age at death)	52% women
Uretsky et al. [26]	Rush University Alzheimer’s Disease Center (ROS and MAP cohorts), IL, US	292 (186 at AD dementia and 54 at prodromal stages, 27 cognitively unimpaired, 21 mixed with AD dementia, 4 non-AD dementias; all with neuropathologic AD confirmation)	Not reported but likely mostly amnestic AD and prodromal AD cases	From 71 to 103 years (at death)	74% women
Smirnov et al. [27]	UCSD- Shiley- Marcos Alzheimer’s Disease Research Center, CA, US	121 (102 at AD dementia and 10 at prodromal AD stages, 9 other non-AD dementias; all with neuropathologic AD confirmation)	Not reported	77 years (average age at death)	36% women
In vivo* tau PET studies*					
Schwarz et al. [29]	Clinical trial (NCT 02,016,560), US	75 (28 at AD dementia and 47 at prodromal AD stages)	Not reported	From 50 to 95 years (at PET scanning)	52% women
Schwarz et al. [30]	ADNI-2, US and Canada	98 (46 healthy controls, 42 MCI, and 10 AD; among them, 52 were amyloid-beta positive, unknown status in 4 cases)	Amnestic AD and MCI cases	75 years (average age at PET scanning)	42% women
Whitwell et al. [11]	Mayo Clinic, Rochester, MN, US	62 (all at the AD dementia stage)	Typical and atypical AD, including posterior cortical atrophy, logopenic aphasia, and behavioral/dysexecutive AD	From 57 to 80 years (at PET scanning)	53% women
Charil et al. [6]	Clinical trial (NCT 02,016,560), US	45 (22 at AD dementia and 23 at prodromal AD stages; all amyloid-beta positive)	Not reported	From 50 to 92 years (at PET scanning)	56% women
Mohanty et al. [8]	ADNI-2, US and Canada	84 (30 at AD dementia and 54 at prodromal AD stages; all amyloid-beta positive)	Amnestic AD and prodromal AD cases	From 55 to 91 years (at PET scanning)	49% women
Palleis et al. [32]	Ludwig-Maximilians-University, Germany	11 patients with Corticobasal Syndrome, 10 of them with underlying AD pathology (biomarker-based)	Corticobasal Syndrome	76 years (average age at PET scanning)	73% women
Rullmann et al. [33]	GII4T: Leipzig and Munich, Germany, and New Haven, CT, US	38 at AD dementia; all amyloid-beta positive	Not reported	69 years (average age at PET scanning)	55% women
Krishnadas et al. [34]	AIBL, Australia	151 (84 at AD dementia and 67 at prodromal AD stages; all amyloid-beta positive)	Mostly amnestic AD and prodromal AD cases	73 years (average age at PET scanning)	48% women
Young et al. [12]	Anti-Amyloid Treatment in Asymptomatic AD study (A4), ﻿ADNI, HABS, and the Wisconsin Registry for Alzheimer’s Prevention and the Wisconsin Alzheimer’s Disease Research Center	392 (cognitively unimpaired, all amyloid-beta positive). The article also includes 55 amyloid-beta negative participants who were excluded for our current study	All cases are cognitively unimpaired	72 years (average age at PET scanning)	57% women
Toledo et al. [31]	ADNI, US and Canada	282 (AD dementia, prodromal AD, and cognitively unimpaired, amyloid-beta positive). The article also includes 214 amyloid-beta negative participants who were excluded for our current study	Amnestic AD and prodromal AD cases	From 61 to 83 years (at PET scanning)	52% women

*FL* Florida; *US* United States, *MN* Minnesota, *UCSF-MAC* University of California San Francisco—Memory and Aging Center, *CA* California, *NCT* National Clinical Trial number, *ADNI* Alzheimer's Disease Neuroimaging Initiative, *AD* Alzheimer's disease, *AIBL* Australian Imaging Biomarkers and Lifestyle study, *GII4T* German Imaging Initiative for Tauopathies, *HABS* Harvard Aging Brain Study, *MCI* mild cognitive impairment, *PET* positron emission tomography, *UCSD* University of California, San Diego

Below we include a narrative description of studies providing data on our main question: “*Can AD cases have NFT in the association cortex while completely sparing the hippocampus (or the entorhinal cortex)?*”.

In Murray et al. [7], 11% (97/889) of the cases belonged to the hippocampal sparing AD subtype. All hippocampal sparing AD cases were at Braak stages > IV, implying hippocampal involvement. Hence, none of the AD cases that had NFT in the association cortex had the hippocampus completely spared of NFT. A recent study included these cases in a larger and updated cohort of 1 361 AD cases at Braak stages > IV [4]. The reported frequency of hippocampal sparing AD cases was 13% (175/1361). Due to the partial overlap between these two cohorts, we will consider the seminal and key study of Murray et al. [7] for our analysis in Table 3.

**Table 3:** Frequency of the hippocampal-sparing AD subtype across studies and from our original data

A	Data from the systematic review: postmortem studies					
Study	Murray et al. 2011 [7]	Whitwell et al. [9]	Petersen et al. [25]	Corder et al. [28]	Uretsky et al. [26]	Smirnov et al. [27]
Data modality	Postmortem (NFT count)	Postmortem (NFT count)	Postmortem (NFT count)	Postmortem (NFT count)	Postmortem (NFT count)	Postmortem (NFT count)
Braak’s tau NFT stage	V or VI	V or VI	V or VI	I to VI	V or VI	V or VI
Subtyping algorithm	Murray^b^	Murray^b^	Murray^b^	Data-driven (GoM)	Approximation of Murray^b^	Approximation of Murray^b^
Sample size	889	177	74	249	292	121
Percentage of individuals with hippocampal-sparing AD	11%	11%	7%	–	8%	19%
Percentage of individuals with NFT count or tau PET uptake completely sparing the hippocampus, according to:						
Neuropathologic definition	0%	0%	0%	0%	0%	0%
B	Data from the systematic review: in vivo tau PET studies					
Study	Schwarz et al. [29]	Schwarz et al. [30]	Palleis et al., [32]	Rullmann et al. [33]	Krishnadas et al. [34]	Toledo et al. [31]
Data modality	PET (flortaucipir)	PET (flortaucipir)	PET (PI-2620)	PET (PI-2620)	PET (MK-6240)	PET (flortaucipir)
Braak’s tau NFT stage	0 to VI^a^	0 to VI^a^	Unknown	I to VI^a^	Unknown	Unknown
Subtyping algorithm	Braak staging^a^	Braak staging^a,c^	Tau PET positivity in cortex in conjunction with tau PET negativity in mesial temporal lobe	Braak staging ^a^	Visual inspection based on Murray^b^	Data-driven (Robust collaborative clustering)
Sample size	75	98	10	38	151	
Percentage of individuals with hippocampal-sparing AD	5%	15%^c^ 7%^c^ 1%^c^	55%	18%	18%	Identified but frequency not reported
Percentage of individuals with NFT count or tau PET uptake completely sparing the hippocampus, according to:						
Neuropathologic definition	5%	15% ^c^ 7% ^c^ 1% ^c^	55%	18%	18%	Identified but frequency not reported
C	Re-analysis from published studies			New data using the ADNI cohort		
Study	Whitwell et al. [11]	Charil et al. [6]	Young et al. [12]	Mohanty et al. [8] (Byun’s algorithm15—hippocampus)	Mohanty et al. [8] (Charil’s algorithm6—hippocampus)	Mohanty et al., [8] (Risacher’s algorithm20—hippocampus)
Data modality	PET (flortaucipir)	PET (flortaucipir)	PET (flortaucipir)	PET (flortaucipir)	PET (flortaucipir)	PET (flortaucipir)
Braak’s tau NFT stage	Unknown	V or VI ^a^	Unknown	Unknown	Unknown	Unknown
Subtyping algorithm	Data-driven (K-means clustering)	Murray^d^	Approximation of Murray^b^	Byun^e^	Murray^d^	Murray^f^
Sample size	62	45	392	84	84	84
Percentage of individuals with hippocampal-sparing AD	34%	13%	9%	21%	10%	11%
Percentage of individuals with NFT count or tau PET uptake completely sparing the hippocampus, according to:						
Neuropathologic definition	**–**	**–**	**–**	**–**	**–**	**–**
*‘Accuracy-based cut point’*	3% ^g^	9%	6%	0%	0%	0%
*‘* + *1SD cut point’*	34% ^g^	13%	9%	21%	6%	7%
*‘10% cut point’*	34% ^g^	13%	9%	21%	6%	7%
*‘Schöll cut point’*	5% ^g^	11%	7%	0%	0%	0%
*‘Maass cut point’*	2% ^g^	2%	1%	0%	0%	0%

^a^Braak staging [10] was based on tau PET data

^b^Murray’s subtyping algorithm is explained in Murray et al. [7]. Briefly, the ratio of the hippocampal to cortical NFT counts was split at the 25th and 75th percentiles of the sample distribution. At a first step, individuals with the ratio < 25% were assigned to hippocampal-sparing AD, those with ratio > 75% were assigned to limbic-predominant AD, and all the rest were assigned to typical AD. At a second step, individuals were re-classified based on median NFT counts in hippocampus and cortex. Brain areas considered in Murray’s subtyping algorithm are hippocampus (CA1 and subiculum), superior temporal cortex, middle frontal cortex, and inferior parietal cortex

^c^Schwarz et al. [30] used three classification schemes for tau staging. The first scheme is the same than in Schwarz et al. [29] and was designed to mimic Braak staging [10] as closely as possible. The second scheme was a simplified version of the first scheme using fewer and larger ROIs located in medial, lateral, and superior temporal lobes and in the primary visual cortex. The third scheme was even simpler than the first two schemes and used lobar ROIs: temporal, frontal, parietal, and occipital. Percentages are reported in order of appearance (first, second, and third algorithm)

^d^The subtyping algorithm in Charil et al. [6] is the same as in Murray et al. [7] with a minor difference in relation to the brain areas considered in the algorithm, substituting CA1 and subiculum areas of the hippocampus for anterior-most position (head) of hippocampus or entorhinal cortex (both methods were tested, for results from the method using entorhinal cortex please see Additional file 1: Table S5). Superior temporal cortex, middle frontal cortex, and inferior parietal cortex are the same as in Murray et al. [7]

^e^Based on the original algorithm described in Byun et al. [15] regional tau PET uptake measures were adjusted for age using multiple linear regression based on a normative group of amyloid-negative healthy controls from ADNI. Using the normative group, Z-scores of hippocampal/entorhinal cortex, frontal, temporal, and parietal regions were calculated and classified as abnormal when Z-score > 1.0. Subtypes were then determined exactly as in Byun et al. [15] Brain areas considered in Byun’s algorithm are the same as in Murray et al. [7] with a minor difference with substituting CA1 and subiculum areas of the hippocampus for hippocampus or entorhinal cortex (both methods were tested, please see Additional file 1: Table S5). Superior temporal cortex, middle frontal cortex, and inferior parietal cortex are the same as in Murray et al. [7]

^f^The subtyping algorithm in Risacher et al.^20^ is the same as in Murray et al.,^7^ with a minor difference in relation to the brain areas considered in the algorithm, substituting CA1 and subiculum areas of the hippocampus for hippocampus or entorhinal cortex (both methods were tested, please see Additional file 1: Table S5), and extending the cortical regions to include middle frontal cortex, inferior frontal cortex, superior temporal cortex, inferior parietal cortex, superior parietal cortex, and supramarginal cortex

^g^Based on the entorhinal cortex instead of the hippocampus. We estimated the approximate proportion of cases who had NFT in the association cortex while completely sparing the hippocampus, based on pooling of all the tau PET data independently of cut point and subtyping method. To do this, each cell from tau PET studies in the *“Percentage of individuals with NFT count or tau PET uptake completely sparing the hippocampus”* section of the table was treated as an independent study. The total of cases with *cortical tau PET uptake completely sparing the hippocampus* was computed (*n* = 372) and divided by the total number of cases included in the studies (*N* = 5583). The resulting proportion is 8%. GoM = Grade of Membership analysis; AD = Alzheimer’s disease; ADNI = Alzheimer's Disease Neuroimaging Initiative; NFT = neurofibrillary tangles; SD = standard deviation; pc = percentile; PET = positron emission tomography

Whitwell et al. [9] applied Murray’s algorithm on an independent sample of 177 cases with a neuropathologic diagnosis of AD, all of whom at Braak stages > IV. The percentage of hippocampal sparing AD was 11% (19/177). As in Murray et al. [7], all hippocampal sparing AD cases were at Braak stages > IV, implying hippocampal involvement. Hence, none of these cases had the hippocampus completely spared of NFT. Strikingly, the neuropathologically-defined hippocampal sparing AD cases showed complete sparing of the hippocampus in terms of atrophy as assessed by MRI data (at the group level). This demonstrates that AD cases with a lower proportion of NFT counts in the hippocampus than in the association cortex do not show any evidence of reduced hippocampal volume (or entorhinal thinning) on MRI when compared to healthy controls. A recent study used the same cohort but only focused on cases with non-amnestic AD presentations at Braak stages IV to VI (N = 36) [24]. The reported frequency of hippocampal sparing AD cases was 31% (11/36), showing the higher frequency of this subtype in atypical AD. Due to the overlap between these two cohorts, we will consider the much larger study of Whitwell et al. [9] for our analysis in Table 3.

Petersen et al. [25] also applied Murray’s subtyping algorithm on 74 cases with a neuropathologic diagnosis of AD, all of them at Braak stages > IV. The group average for subtype classification was derived from the 74 pure AD cases who lacked co-existing pathology, which may affect thresholds. Clinically, the cases spanned from typical AD to various atypical/non-amnestic syndromes. The percentage of hippocampal sparing AD was 7% (5/74). None of the cases who had NFT in the association cortex had the hippocampus completely spared of NFT.

Uretsky et al. [26] used an approximation of the Murray’s subtyping algorithm on 292 cases with a neuropathologic diagnosis of AD, all of them at Braak stages > IV, but with clinical diagnoses including AD dementia, prodromal AD, preclinical AD, mixed AD dementia, and non-AD dementias. The percentage of hippocampal sparing AD was 8% (22/292). None of the cases who had NFT in the association cortex had the hippocampus completely spared of NFT.

Smirnov et al. [27] also used an approximation of Murray’s subtyping algorithm on 121 cases with a neuropathologic diagnosis of AD, all of them at Braak stages > IV, but with clinical diagnoses including AD dementia, prodromal AD, and non-AD dementias. The percentage of hippocampal sparing AD was 19% (23/121). None of the cases who had NFT in the association cortex had the hippocampus completely spared of NFT.

In Corder et al. [28], all cases with NFT counts in the association cortex also had NFT counts in CA1 and subiculum. Hence, as per the reported data, none of the AD cases who had NFT in the association cortex had the hippocampus completely spared of NFT. This analysis was based on 249 cases. A total of 159 cases had a neuropathologic diagnosis of AD, and Braak stages ranged from I to VI in the whole cohort.

These eight neuropathologic studies support the “*cortical predominance*” and “*cortical precedence*” hypotheses (Fig. 2b, c). However, except for Corder et al. [28], these studies could not really test for the “*distinct cortical*” hypothesis (Fig. 2d), because they all included cases at Braak stage IV [24] or > IV [4, 7, 9, 25–27].

Schwarz et al. [29] used the tau PET tracer flortaucipir to assess NFT in vivo. In their study, 5% (4/75) of the amyloid-positive prodromal AD or AD dementia participants revealed flortaucipir uptake in the association cortex while completely sparing the hippocampus (normal flortaucipir uptake in the hippocampus). However, among the cortical regions they tested, these four participants showed abnormal flortaucipir uptake in the transentorhinal cortex.

In a later publication, Schwarz et al. [30] used the tau PET tracer flortaucipir in the ADNI cohort, including 46 cognitively unimpaired participants [19 amyloid-positive], 42 mild cognitive impairment (MCI) participants [24 amyloid-positive], and 10 AD dementia participants [9 amyloid-positive]. The authors tested three classification schemes for tau staging. The first scheme, which was designed to mimic Braak staging as closely as possible, showed that 14% (14/98) of the participants had an abnormal flortaucipir uptake in the association cortex while completely sparing the hippocampus. Three of these participants showed an abnormal flortaucipir uptake in the transentorhinal cortex. The second scheme, which was a simplified version of the first scheme using fewer and larger ROIs, showed that 7% (7/98) of the participants had an abnormal flortaucipir uptake in the association cortex while completely sparing the medial temporal lobe. The third scheme, which was even simpler than the first two schemes and used lobar ROIs, showed that only 1% (1/98) of the participants had an abnormal flortaucipir uptake in non-temporal lobes while completely sparing the temporal lobe. However, we cannot exclude that some of these cases are amyloid-negative since the data was not reported stratified by amyloid status.

Whitwell et al. [11] performed a clustering analysis on flortaucipir uptake in the entorhinal cortex and a ROI including 17 neocortical regions, on 62 amyloid-positive AD dementia participants. The authors reported that 34% (21/62) of their participants were classified as low entorhinal and high cortical flortaucipir uptake, consistent with our definition of hippocampal sparing AD.

Charil et al. [6] applied Murray’s subtyping algorithm on tau PET data using the flortaucipir tracer. All participants were amyloid-beta positive: 23 were at the prodromal AD stage and 22 were at the AD dementia stage. The authors reported that 13% (6/45) of the participants were classified as hippocampal sparing AD. However, as in Murray et al. [7], all hippocampal sparing AD cases were at Braak stages > IV based on tau PET, implying hippocampal involvement. Hence, none of these cases had the hippocampus completely spared of NFT.

Young et al. [12] used an approximation of Murray’s subtyping algorithm on tau PET data using the flortaucipir tracer. All participants were amyloid-beta positive and cognitively unimpaired. The authors reported that 9% (36/392) of the participants had a divergent cortical tau pattern, roughly consistent with the hippocampal sparing AD subtype.

Toledo et al. [31] used a data-driven method on the tau PET tracer flortaucipir. All participants were amyloid-beta positive, including individuals at the AD dementia stage, prodromal AD, and cognitively unimpaired. Their data-driven method identified clusters within a gradient of increasing tau PET uptake (cluster 1: *n* = 181; cluster 2: *n* = 75; cluster 3: *n* = 16; cluster 4: *n* = 10). The largest cluster, cluster 1, was subclustered in a sensitivity analysis, demonstrating the existence of a subtype consistent with hippocampal sparing AD. However, the frequency of this subtype was not reported.

Palleis et al. [32] used a different tau PET tracer, 18F-PI-2620. The authors included 45 patients with a Corticobasal Syndrome, of whom 10 had underlying AD pathology based on biomarkers. Visual inspection of the data reported by the authors reveals that 60% (6/10) of the participants had tau PET positivity in cortical areas in conjunction with tau PET negativity in mesial temporal lobe, which is consistent with hippocampal sparing AD.

Rullmann et al. [33] also used the tau PET tracer 18F-PI-2620. The authors assessed 38 participants with AD dementia who were amyloid-beta positive. The authors reported that 18% (7/38) of the participants were classified with the hippocampal sparing AD subtype. The authors used the same method than in Schwarz et al. [29], so that these participants revealed 18F-PI-2620 uptake in the association cortex while completely sparing the hippocampus (normal 18F-PI-2620 uptake in the hippocampus). However, the authors did not report whether 18F-PI-2620 uptake also spared the transentorhinal cortex.

Krishnadas et al. [34] used a third different tau PET tracer, 18F-MK-6240. All participants were amyloid-beta positive: 67 were at the prodromal AD stage and 84 were at the AD dementia stage. The authors reported that 18% (27/151) of the participants were classified as hippocampal sparing AD, although the authors stated that 18F-MK-6240 tracer uptake was no or minimal on visual inspection.

Hence, the results from these tau PET studies serve as a preliminary support to the “*distinct cortical*” hypothesis (Fig. 2d). To further test the “*distinct cortical*” hypothesis, we re-analyzed the data available from Whitwell et al. [11], Charil et al. [6], and Young et al. [12], and investigated the ADNI cohort so as to identify hippocampal sparing AD participants who had normal tau PET uptake values in the hippocampus or entorhinal cortex (see next section).

### Original data

Table 3 shows our re-analysis of the data reported in Whitwell et al. [11], Charil et al. [6], and Young et al. [12].

In Whitwell et al. 11, we observed that two out of their 21 hippocampal sparing AD participants had a pattern of flortaucipir uptake completely sparing the entorhinal cortex, according to the *‘accuracy-based cut point’* (3%, 2/62, of the whole cohort). The *‘* + *1SD cut point’* and ‘*10% cut point*’ revealed that all their 21 hippocampal sparing AD participants had a pattern of flortaucipir uptake completely sparing the entorhinal cortex (34%, 21/62, of the whole cohort). The percentages for the *‘Schöll cut point’* and *‘Maass cut point’* are 5% and 2%, respectively (Table 3).

In Charil et al. [6], we observed that four out of their six hippocampal sparing AD participants had a pattern of flortaucipir uptake completely sparing the hippocampus, according to the *‘accuracy-based cut point’* (9%, 4/45, of the whole cohort). The *‘* + *1SD cut point’* and *‘10% cut point’* revealed that all their hippocampal sparing AD participants had a pattern of flortaucipir uptake completely sparing the hippocampus (13%, 6/45, of the whole cohort). The percentages for the *‘Schöll cut point’* and *‘Maass cut point’* are 11% and 2%, respectively (Table 3).

In Young et al. 12, we observed that 23 out of their 36 hippocampal sparing AD participants had a pattern of flortaucipir uptake completely sparing the medial temporal lobes, according to the *‘accuracy-based cut point’* (6%, 23/392, of the whole cohort). The *‘* + *1SD cut point’* and *‘10% cut point’* revealed that all their 36 hippocampal sparing AD participants had a pattern of flortaucipir uptake completely sparing the medial temporal lobes (9%, 36/392, of the whole cohort). The percentages for the *‘Schöll cut point’* and *‘Maass cut point’* are 7% and 1%, respectively (Table 3).

Finally, we produced new data using the ADNI cohort. In our recent study by Mohanty et al. [8], we applied three subtyping algorithms on tau PET data (flortaucipir) from the ADNI cohort. The algorithm based on Byun et al. [15] revealed that 21% (18/84) of the amyloid-positive prodromal AD or AD dementia participants belonged to the hippocampal sparing AD subtype. According to this algorithm originally based on the *‘* + *1SD cut point’*, all 18 hippocampal sparing AD participants had a pattern of flortaucipir uptake completely sparing the hippocampus. The percentages for the alternative cut points are shown in Table 3 and range from 0 to 21%. When we replicated Charil et al. [6] and Risacher et al. [20] algorithms in the ADNI cohort, we found that 10% (8/84) and 11% (9/84) of the amyloid-positive prodromal AD or AD dementia participants belonged to the hippocampal sparing AD subtype, respectively. However, the algorithms by Charil et al. [6] and Risacher et al. [20] do not completely exclude that hippocampal sparing AD participants can have abnormal flortaucipir uptake values in the hippocampus. The reason for that is that these two algorithms define hippocampal sparing AD as the 25% of cases with highest flortaucipir uptake in the association cortex as compared with flortaucipir uptake in the hippocampus. Hence, using Charil et al. [6] and Risacher et al. [20] algorithms, we determined abnormal levels of flortaucipir uptake using the cut points described in Table 1. We found that no participant (0%, 0/84) had a pattern of flortaucipir uptake completely sparing the hippocampus when applying the *‘accuracy-based cut point’*. When applying the *‘* + *1SD cut point’* and *‘10% cut point’*, 6% (5/84) and 7% (6/84) of the participants had a pattern of flortaucipir uptake completely sparing the hippocampus in Charil et al. [6] and Risacher et al. [20] algorithms, respectively. Percentages for the *‘Schöll cut point’* and *‘Maass cut point’* were 0% (Table 3). All the results in this paragraph come from subtyping based on the association cortex and the hippocampus. As a control, we did the subtyping based on the association cortex and the entorhinal cortex and we observed very similar results (Additional file 1: Table S5).

In summary, independently of the subtyping algorithm and cohort, several cut points consistently identified participants who had NFT in the association cortex while the hippocampus (or the entorhinal cortex) was completely spared of NFT, as revealed by tau PET. However, the more conservative cut points (*‘Accuracy-based cut point’*, *‘Schöll cut point’*, and *‘Maass cut point’*) found a lower proportion or failed to find hippocampal sparing AD participants in some analyses.

## Discussion

In this study we addressed the question of whether neuropathology and in-vivo tau PET can identify AD cases with NFT in the association cortex while completely sparing the hippocampus (or entorhinal cortex). Our findings suggest that those cases can be identified antemortem, but the ability to detect them depends on how the hippocampal sparing AD subtype is defined and what data modality and cut points are used to assess tau pathology. This finding reflects the importance of reaching a consensus in the field with regard to how to operationalize biological subtypes of AD in future studies [35].

Several in-vivo studies provide supportive evidence of tau accumulating in the association cortex while completely sparing the hippocampus [8, 11, 22, 29, 36]. However, these cases are extremely rare in AD and, so far, they have only been detected by tau PET imaging. In the eight neuropathologic studies reviewed in the current study [4, 7, 9, 24–28], we did not find any individual case with NFT in the association cortex while completely sparing the hippocampus. However, six of those studies included cases at Braak stages > IV, and one study at Braak stages from > III, implying hippocampal involvement as limbic regions are considered to be affected by Braak III [10]. Of particular note, the successful identification and creation of the neuropathologic algorithm that first operationally defined hippocampal sparing AD required the use of NFT counts derived from review of thioflavin-S fluorescent staining [7, 25]. Phospho-tau markers (e.g. AT8) readily recognize early aspects of tangle maturity and may reveal tau pathology that does not entirely correspond to neuronal death [37]. Apart from the design of those studies, it is possible that neuropathologic studies have a lower potential to identify AD cases with NFT in the association cortex while completely sparing the hippocampus. One reason for this is that neuropathologic studies tend to include older individuals at advanced stages of the disease. Braak and Del Tredici [38] showed that in their cohort of 2366 non-selected autopsy cases, virtually all cases had NFT in hippocampus at age 80 and above. The frequency of NFT in hippocampus was between 30 and 85% in the age range from 30 to 79 years. Hence, the chance of finding hippocampal sparing cases is very low and, if any, that chance would be higher when assessments are done in individuals below the age of 60 [38]. Indeed, many of the hippocampal sparing AD cases in Murray et al. [7] had their disease onset before the age of 60.

In contrast, the possibility of PET imaging to assess tau deposition in vivo at younger ages and earlier disease stages is expected to increase the potential to identify hippocampal sparing AD cases. This is what our current study also suggest. Our tau PET analyses show that when pooling all the data together, 372/5 583 cases (8%, see legend of Table 3 for further details) had tau PET uptake in the association cortex while completely sparing the hippocampus [6, 8, 11, 12, 29–34]. The important question is whether these cases will fit in the *“cortical precedence”* hypothesis, that is, they start with NFT in the association cortex but will accumulate NFT in hippocampus as the disease progresses; or rather, these cases fit in the *“distinct cortical”* hypothesis, that is, they start with NFT in the association cortex and will not accumulate NFT in hippocampus during the entire progression of the disease. Unfortunately, there is no data at present that can resolve this question because the participants should have been scanned with tau PET from negative tau stage to earliest tau positive stages, up to death. As for neuropathologic studies, we urgently need subtyping studies on datasets including participants ranging from Braak stage 0 to VI.

The main concern in tau PET studies is that the ability to detect hippocampal sparing AD may depend on the cut points used, provided that any kind of technical issue was successfully excluded (e.g., low tau PET uptake due to technical issues, variation related to partial volume corrections, etc.). Indeed, this problem is not exclusive of tau PET studies but is a generalized problem in Medicine and Science when trying to determine abnormality in any measure, modality, or population [39, 40]. To circumvent this, we applied five alternative cut points. We included the increasingly used *‘accuracy-based cut point’* of 1.33 for flortaucipir [14]. However, lenient and conservative versions of this cut-point exist [16], which will influence individuals’ belonging to different subtypes [8]. Further, the 1.33 cut-point was established for a meta-ROI region, while a cut point for flortaucipir uptake in the hippocampus or entorhinal cortex has not been completely agreed upon yet. We thus computed two other common cut points using the publicly available ADNI data [13], including the ‘+*1SD cut point*’ [15] and the ‘*10% cut point*’ [16]; and we added two more cut points that are popular in the field (i.e., *‘Schöll cut point’*, and *‘Maass cut point’*) [18, 19].

We found that independent of the subtyping algorithm and cohort used several cut points identified participants who had NFT in the association cortex while completely sparing the hippocampus or the entorhinal cortex, as revealed by tau PET. Indeed, several hippocampal sparing participants had normal flortaucipir uptake values in hippocampus/entorhinal cortex far from any of the cut points, hence highlighting the ability of tau PET to identify these cases. However, the more conservative cut points (i.e., *‘accuracy-based cut point’*, *‘Schöll cut point’*, and *‘Maass cut* point’) did not detect these cases, at least in the ADNI data used in our analyses. Hence, our current study illustrates the importance of developing and agreeing upon the cut points for specific brain regions that are relevant for performing Braak staging in vivo, and for scientific questions such as identifying subtypes of AD. Further, the cut points should also be tested and validated in different large unselected cohorts, in addition to research cohorts with strict selection criteria like ADNI [41].

The existence of hippocampal sparing cases with complete sparing of the hippocampus/entorhinal cortex is supported by recent data suggesting alternative ways of NFT spread in diseases such as dementia with Lewy bodies (DLB). Flortaucipir uptake in DLB primarily involves the posterior cortical regions, sparing hippocampus/entorhinal regions [42–47]. Although more research is needed to fully understand the meaning of flortaucipir uptake in non-AD tauopathies [21], this atypical pattern of flortaucipir uptake in DLB matches perfectly with the characteristic hypometabolic FDG PET pattern in DLB involving the parietal and occipital cortex [48], as well as with the location of white matter hyperintense lesions [49, 50], pattern of white matter disruption [51], and reduced blood perfusion [52], all of which predominantly involve posterior brain regions. Interestingly, we applied our AD subtyping algorithm on 333 DLB participants from 15 centers across Europe and showed that hippocampal sparing was the most common pattern of atrophy in DLB [53]. This and some other data [9, 54, 55] led us to propose that comorbid Lewy body pathology may be associated with the hippocampal sparing subtype of AD [1]. However, another cohort reported a higher frequency of Lewy body pathology in limbic predominant and typical AD [7], so the association between Lewy body pathology and AD subtypes still needs to be elucidated. We recently found that the volume of the cholinergic basal forebrain declines more slowly and response to cholinergic treatment seemed to be better in hippocampal sparing AD [56]. DLB and AD patients with less hippocampal atrophy respond well to cholinesterase inhibitors [57–59]. Supporting neuropathologic observation of lower NFT counts in nucleus basalis of Meynert in hippocampal sparing AD [4], we suggested that an intact hippocampus responding to cholinergic input may be an explanation for good response to cholinergic treatment in DLB and hippocampal sparing AD [56]. Whether a common pattern of brain atrophy or increased Lewy body pathology in hippocampal sparing AD, or both, is the reason for this finding needs to be clarified. It is possible that a proportion of participants with abnormal flortaucipir uptake values in the association cortex but completely sparing hippocampus/entorhinal regions are indeed individuals with Lewy body disease diagnosed as AD, as opposed to AD individuals with comorbid Lewy body pathology. A finding supporting this possibility is that AD cases with comorbid Lewy body disease likely have NFT in the hippocampus [7], as typical AD and limbic predominant AD were reported to have the highest proportion compared to hippocampal sparing AD [4, 7, 60].

MRI studies have consistently identified hippocampal sparing AD cases [1, 3]. However, MRI studies assess variation in regional brain atrophy. While MRI can reliably track neuropathologically-defined AD subtypes [9], neuropathologies other than NFT also contribute to the variation in regional brain atrophy. Hence, a proportion of participants classified as hippocampal sparing AD in MRI studies without neuropathologic confirmation may not have any NFT in the association cortex but rather have other neuropathologies. Similarly, a proportion of participants classified as typical AD on MRI studies may have NFT only in the association cortex with hippocampal atrophy coming from pathologies such as hippocampal sclerosis, TDP-43, or cerebrovascular disease [61, 62]. Some support for this idea can be seen in our recent publications by Mohanty et al. [8, 63, 64]. Further, the temporal gap between NFT accumulation and subsequent brain atrophy may be a confounder of hippocampal sparing AD in MRI studies. In other words, a proportion of participants classified as hippocampal sparing AD in MRI studies without neuropathologic confirmation may have a typical pattern of NFT accumulation. For instance, Ossenkoppele et al. [65] recently showed that their MRI-defined hippocampal sparing AD subtype had elevated flortaucipir uptake in the entorhinal cortex, in addition to prominent flortaucipir uptake in the association cortex. We also showed that participants with the MRI-defined hippocampal sparing AD subtype can be classified as typical AD or even as limbic predominant AD when using flortaucipir data [8]. In keeping with the discussion about neuropathologic pathways, the only study to date that has applied longitudinal clustering on MRI data showed that the hippocampal sparing subtype can eventually develop a typical AD pattern of atrophy, hence involving hippocampus/entorhinal cortex [66]. This would support the “*cortical precedence*” hypothesis but analyses at the individual level could confirm whether some cases could fit in the “*distinct cortical”* hypothesis instead.

Future perspectives include accumulation of more studies using second-generation tau PET tracers, implementation of the centiloid approach to determine abnormality in tau PET, and expansion of current subtyping rationale to include subcortical nuclei such as nucleus basalis of Meynert and locus coeruleus. Most of the reviewed tau PET subtype studies used flortaucipir, while we identified two recent studies using the 18F-PI-2620 tracer and one using the 18F-MK-6240 tracer. While flortaucipir is excellent in depicting tau pathology in regions comprising late Braak stages, its performance for early tau stages is more limited [67, 68]. Second generation tau PET tracers such as 18F-PI-2620 and 18F-MK-6240 seem more sensitive to early tau pathology [67], which could help to identify hippocampal sparing cases. This idea is supported by our current analyses (see Table 3), but more second-generation tau PET tracer studies are needed to confirm this finding. Although, head-to-head studies including several tau PET tracers are scarce, recent research shows variation in the regional retention of flortaucipir and second-generation tau PET tracers (RO-948, MK6240) [68, 69]. A prospect for the future is to understand the performance of different tau PET tracers in hippocampal sparing cases, and atypical AD cases in general. Further, cut points are somewhat arbitrary. For that reason, we investigated five complementary cut points. Similar to amyloid PET, the centiloid approach is currently being promoted in the field of tau PET, so that a single standardized scale can be used [17]. Future studies should test potential advantages of the centiloid approach for subtyping. Finally, data suggest that the locus coeruleus and nucleus basalis of Meynert may be the earliest sites for NFT accumulation, preceding NFT in limbic/cortical brain areas [4, 38, 70, 71]. The field of biological subtypes of AD has not yet implemented nucleus basalis of Meynert and locus coeruleus in subtyping algorithms and so, we focused our current study on limbic/cortical NFT.

A limitation of our study is that the percentage of hippocampal sparing as determined by the Murray’s algorithm in [4, 6, 7, 9, 12, 24–27] is partly influenced by the definition of hippocampal sparing AD in that algorithm (based on the 25th percentile). Nonetheless, our study shows that the percentages obtained by the Murray’s algorithm seem to be in the range of percentages obtained by the other investigated algorithms. The percentage of hippocampal sparing AD also varied when using conservative or lenient cut points for tau PET. Future studies could use visual rating of tau PET to complement our current approach.

This study demonstrates that tau PET can identify hippocampal sparing cases with NFT completely sparing the hippocampus. We cannot exclude that neuropathology also has the potential to identify those cases, but 7 out of the 8 neuropathologic studies identified in our systematic review exclusively analyzed cases at Braak stage IV or higher, which by definition have NFT in the hippocampus. Future subtyping studies should include participants ranging from Braak stage 0 to VI. Further, we introduced three hypotheses of NFT spread in hippocampal sparing AD. Future work needs to investigate the temporal trajectories of NFT accumulation in hippocampal sparing AD, in vivo, by using longitudinal tau PET data in amyloid-positive participants along the AD continuum. This will allow for elucidating the etiology of hippocampal sparing AD as NFT initiating in association cortex while completely sparing the hippocampus (the *“distinct cortical”* hypothesis), or whether NFT in both the association cortex and hippocampus are observed at advanced Braak stages (the *“cortical predominance”* or *“cortical precedence”* hypotheses). The recent studies by Vogel et al. [22, 72] and Franzmeier et al. [36] based on cross-sectional tau PET data showed that, although rare, some participants show epicenters of tau spreading alternative to the entorhinal cortex. For instance, in one of the subtypes resembling hippocampal sparing AD in Vogel et al. [22], tau seemed to progress rapidly from parietal to lateral temporal and frontal regions, sparing the medial temporal lobes across the entire disease progression [22]. This subtype would fit in the *“distinct cortical”* hypothesis and may thus represent hippocampal sparing cases with NFT completely sparing the hippocampus.

Altogether, based on the accumulating data we suggest that there are perhaps two independent pathways of limbic/cortical tau spread that initiates with subthreshold levels of biomarker-measured pathology, converting to a minimal degree of pathology in either hippocampus/entorhinal cortex or association cortex (i.e., minimal tau subtype in PET studies, or minimal atrophy subtype in MRI studies [8]). From that initial timepoint, the most common pathway would be the spread of NFT as encapsulated in Braak staging [10]. The less common alternative pathway would be the spread of tau initiating and progressively accumulating in the association cortex without any involvement of the hippocampus and/or entorhinal cortex (the *“distinct cortical”* hypothesis), or with involvement of the hippocampus and/or entorhinal cortex as the disease progresses (the *“cortical predominance”* or *“cortical precedence”* hypotheses) (Fig. 2b-d). In this paragraph we are mostly discussing limbic/cortical stages of NFT spreading, since it was suggested that tau pathology could also start in nucleus basalis of Meynert [4, 38, 70], or even start independently at several sites in parallel [70].

We encourage that future studies report NFT counts or tau PET uptake levels in individual cases, so that the reader can evaluate the certainty for a hippocampal sparing case to belong to that subtype versus how cut points may influence that classification. Also, future neuropathologic studies could investigate NFT counts in the association cortex in Braak stage 0 or I (in cases with no NFT in hippocampus). All these suggestions may help to continue moving the field forward, and our current study illustrates the importance of harmonizing the methods for operationalization of biological AD subtypes across studies [8, 35].

## Supplementary Information

**Additional file 1: Table S1. **Search strategy. **Table S2.** Strategies followed to reduce the risk of bias. **Table S3.** List of fields covered for the collection of the data (data extraction template). **Table S4.** Reasons for excluding candidate records (inclusion stage in the study-selection flow). **Table S5.** Frequency of the hippocampal-sparing AD subtype – comparison of findings when using hippocampus versus entorhinal cortex for subtyping.

## Data Availability

The datasets used and/or analysed during the current study available from the corresponding author on reasonable request.
