# Supplementary material for: The hippocampal sparing subtype of Alzheimer’s disease assessed in neuropathology and in vivo tau positron emission tomography: a systematic review
Source: Acta Neuropathol Commun. 2022 Nov 14;10:166. doi: 10.1186/s40478-022-01471-z (PMC9664780; doi:10.1186/s40478-022-01471-z)
Supplement: Supplementary file 1 — Additional file 1: Table S1. Search strategy. Table S2. Strategies followed to reduce the risk of bias. Table S3. List of fields covered for the collection of the data (data extraction template). Table S4. Reasons for excluding candidate records (inclusion stage in the study-selection flow). Table S5. Frequency of the hippocampal-sparing AD subtype – comparison of findings when using hippocampus versus entorhinal cortex for subtyping. [file 40478_2022_1471_MOESM1_ESM.docx]

**SUPPLEMENTARY MATERIAL**

**Supplementary Table 1. Search strategy**

| **Search** | **Term 1** | **Term 2** | **Term 3** | **Term 4** | **PUBMED hits** | **WOS**  **hits** | **EMBASE hits** |
| --- | --- | --- | --- | --- | --- | --- | --- |
| *Systematic review from our previous publication* ^1^ *(search in July 2019)* | | | | | | | |
| SEARCH 1 | Alzheimer | subtype | atrophy | - | 55 | 290 | 164 |
| SEARCH 2 | AD | subtype | atrophy | - | 61 | 176 | 96 |
| SEARCH 3 | Alzheimer | heterogeneity | atrophy | - | 95 | 181 | 272 |
| SEARCH 4 | AD | heterogeneity | atrophy | - | 97 | 113 | 183 |
| SEARCH 5 | Alzheimer | atrophy | patterns | subtypes | 37 | 123 | 90 |
| SEARCH 6 | AD | atrophy | patterns | subtypes | 31 | 78 | 50 |
| SEARCH 7 | Alzheimer | MRI | heterogeneity | - | 125 | 110 | 200 |
| SEARCH 8 | AD | MRI | heterogeneity | - | 227 | 76 | 155 |
| SEARCH 9 | Alzheimer | Magnetic Resonance | heterogeneity | - | 114 | 94 | 381 |
| SEARCH 10 | AD | Magnetic Resonance | heterogeneity | - | 221 | 64 | 243 |
| SEARCH 11 | Alzheimer | Magnetic Resonance | subtype | - | 60 | 126 | 232 |
| SEARCH 12 | AD | Magnetic Resonance | subtype | - | 124 | 80 | 143 |
| SEARCH 13 | Alzheimer | PET | heterogeneity | - | 57 | 84 | 169 |
| SEARCH 14 | AD | PET | heterogeneity | - | 101 | 59 | 123 |
| SEARCH 15 | Alzheimer | PET | subtype | - | 28 | 157 | 138 |
| SEARCH 16 | AD | PET | subtype | - | 73 | 92 | 76 |
| SEARCH 17 | Alzheimer | PET | patterns | - | 316 | 848 | 974 |
| SEARCH 18 | AD | PET | patterns | - | 376 | 614 | 749 |
| SEARCH 19 | Alzheimer | subtype | postmortem | - | 42 | 83 | 39 |
| SEARCH 20 | Alzheimer | heterogeneity | postmortem | - | 79 | 47 | 55 |
| SEARCH 21 | Alzheimer | subtype | neurofibrillary tangle | - | 39 | 148 | 56 |
| SEARCH 22 | Alzheimer | heterogeneity | neurofibrillary tangle | - | 85 | 142 | 129 |
| SEARCH 23 | Alzheimer | neuropathological | subtypes | - | 67 | 136 | 159 |
| *Update of systematic review (search in October 22)* | | | | | | | |
| SEARCH 1 | * Searches #13 to #23 as in 2019 , but all duplicates removed in search strategy | | | | 1457 | - | - |

WOS = Web of science; AD = Alzheimer’s disease; MRI = magnetic resonance imaging; PET = positron emission tomography

**Supplementary Table 2. Strategies followed to reduce the risk of bias**

| **Publication bias and reviewer selection bias** | |
| --- | --- |
| 1 | *Systematic review:*  Evidence was rigorously reviewed in order to minimize both publication and reviewer selection bias. |
| 2 | *Manual query of relevant studies:*  Possible publication bias and reviewer selection bias was minimized by supplementing literature review with manual query of relevant studies. |
| 3 | *Examination of missing results or data:*  Selected studies were carefully examined for clues suggesting that there may be missing results or data. |
| **Data availability bias** | |
| 4 | *Assessments were completed independently by more than one researcher:*  Data were sought and assessed by a single researcher. A second researcher was involved every time it was needed for discussion and decisions were taken at consensus. |
| **Methodological quality** | |
| 5 | *CASP:*  Methodological quality was critically appraised with the CASP scale. Assessment was performed by a single researcher and a second researcher was involved if needed. |
| 6 | *PRISMA statement for reporting systematic reviews with meta-analyses:*  The study was performed in accordance with the PRISMA statement, which provides a detailed guideline of a preferred reporting style of transparence. |

**Supplementary Table 3. List of fields covered for the collection of the data (data extraction template)**

| Study | First author |
| --- | --- |
|  | All authors |
|  | Year |
|  | Title of the manuscript |
|  | Journal |
|  | Publication type |
| Research group | Center |
|  | Country |
| Cohort | Center |
|  | Country |
|  | Source of the individuals |
|  | Selection criteria |
|  | Data modality |
|  | Amyloid status |
|  | Braak’s tau NFT stage |
|  | Cut point for interpretation of tau NFT or tau PET binding |
|  | Number of individuals in the AD spectrum |
|  | Number of controls |
|  | Other study groups and number of individuals |
| Subtyping method | Subtyping data (modality) |
|  | Input data (measures) |
|  | Subtyping method |
|  | Methods (specifications) |
|  | Main study outcome |
|  | Group used for subtyping |
| Subtypes | Inclusion of a typical AD subtype? |
|  | Inclusion of a limbic-predominant AD subtype? |
|  | Inclusion of a hippocampal-sparing AD subtype? |
|  | Inclusion of a minimal atrophy or minimal tau AD subtype? |
|  | Other subtype included? |
| Study design | Cross-sectional / longitudinal |
| Subtype, % | Typical AD |
|  | Limbic-predominant AD |
|  | Hippocampal-sparing AD |
|  | Minimal atrophy AD |
|  | Other |
| Subtype, count | Typical AD |
|  | Limbic-predominant AD |
|  | Hippocampal-sparing AD |
|  | Minimal atrophy AD |
|  | Other |

AD = Alzheimer’s disease; NFT = neurofibrillary tangles; PET = positron emission tomography

**Supplementary Table 4. Reasons for excluding candidate records (inclusion stage in the study-selection flow)**

| **Study** | **Type** | **Reason for exclusion from the systematic review** |
| --- | --- | --- |
| Armstrong et al., 2000 ^73^ | postmortem | PCA on SP and NFT showed that the data is distributed rather continuously and distinct subtypes could not be identified |
| Armstrong et al., 1996 ^74^ | postmortem | Five AD groups based on distribution of SP, NFT, and CAA. The cohort includes both familiar and sporadic AD. An hippocampal-sparing subtype can not be easily identified |
| Armstrong and Wood, 1994 ^75^ | postmortem | Five AD groups based on distribution of SP, NFT, and CAA. The cohort includes both familiar and sporadic AD. An hippocampal-sparing subtype can not be easily identified |
| Armstrong and Myers, 1992 ^76^ | postmortem | PCA on pathological data showed that the data is distributed rather continuously and distinct subtypes could not be identified |
| Blennerhassett et al., 2014 ^77^ | postmortem | Clinical subtypes (frontal variant of AD vs. typical AD vs. FTLD) |
| Bondareff et al., 1993 ^78^ | postmortem | Two AD groups based on NFT counts in only one region, i.e. the CA1 region of the posterior hippocampus |
| Coleman et al., 1992 ^79^ | postmortem | Two AD groups based on NFT counts in only one region, i.e. the frontal cortex |
| Cupidi et al., 2010 ^80^ | postmortem | Two AD groups based on the Braak stage, i.e. stage V vs. stage VI. |
| Das et al., 2021 ^81^ | tau-PET + MRI | Six subtypes. An hippocampal-sparing subtype can not be easily identified, and subtypes are based not only on tau-PET but also MRI |
| Dugger et al., 2014 ^82^ | postmortem | No subtypes or groups of AD cases are investigated |
| Franzmeier et al., 2020 ^36^ | tau-PET | The subtyping method (independent component analyses) identifies numerous components and nine subtypes. An hippocampal-sparing subtype can not be easily identified, and data is not reported at the individual level for our original analysis |
| Holzer et al., 1994 ^83^ | postmortem | No subtypes or groups of AD cases are investigated |
| Jeon et al., 2019 ^84^ | tau-PET + PiB-PET + MRI | Three subtypes. Although one of the subtypes seems to resemble the hippocampal-sparing subtype, the subtype is based not only on tau-PET but also PiB-PET and MRI, and data is not reported at the individual level for our original analysis |
| Kovacs et al., 2012 ^85^ | postmortem | Editorial letter, no subtypes or groups of AD cases are investigated |
| Krishnadas et al., 2022 ^86^ | tau-PET | Only four cases reported, who were all amyloid-beta PET negative but had tau PET tracer retention consistent with Braak’s stages V or VI |
| Lowe et al., 2018 ^87^ | tau-PET | Three groups based on hierarchical clustering of regional tau-PET. However, AD and MCI study participants are combined in the analysis and none of the patterns can be related to the hippocampal-sparing subtype |
| Mizuno et al., 2003 ^88^ | postmortem | Two groups of SDAT cases based on count and density of NFT in 4 frontal and temporal regions. An hippocampal-sparing subtype can not be easily identified |
| Mizutani, 1994 ^89^ | postmortem | Three groups of SDAT cases based on pathological information. An hippocampal-sparing subtype can not be easily identified |
| Oh et al, 2022 ^90^ | tau-PET | Two groups of intra temporal versus extra temporal tau PET tracer retention, but the report is only for amyloid-beta PET negative AD dementia patients. There is no mention on whether the extra temporal group had the hippocampus spared, and the groups did not differ in hippocampal volume |
| Singh et al., 2022 ^91^ | tau-PET | No data provided for subtypes other than reports of single cases |
| Stopschinski et al., 2021 ^92^ | postmortem | No subtypes or groups of AD cases are investigated |
| Terry et al., 1987 ^93^ | postmortem | Two AD groups based on disease staging and not potentially distinct subtypes. Data is reported for Braak stage V/VI vs. Braak stages <V |
| Thal et al., 2010 ^94^ | postmortem | Two AD groups based on capillary CAA vs. no capillary CAA. An hippocampal-sparing subtype can not be easily identified |
| Tiraboschi et al., 2004 ^95^ | postmortem | Two AD groups based on disease staging and not potentially distinct subtypes. Data is reported for Braak stage V/VI vs. Braak stages <V |
| Toledo et al., 2016 ^96^ | postmortem | Subtyping is based on alpha-synuclein and AD pathology distribution. An hippocampal-sparing subtype can not be easily identified |
| Vermersch et al., 1992 ^97^ | postmortem | No subtypes or groups of AD cases are investigated |
| Vogel et al., 2019 ^98^ | tau-PET | HC, MCI, and AD study participants are all combined for the cluster analysis, leading to clusters aligning mostly with Braak NFT stages (disease severity) rather than identifying distinct subtypes. An hippocampal-sparing subtype can not be easily identified |
| Vogel et al., 2020 ^72^ | tau-PET | No subtypes or groups of AD study participants are investigated |
| Vogel et al., 2020 ^22^ | tau-PET | Four subtypes. Although one of the subtypes seems to resemble the hippocampal-sparing subtype, data is not reported at the individual level for our original analysis |
| Vogt et al., 1998 ^99^ | postmortem | Five AD groups based on PCA on features measured on neurons in layers III-Va of only one region, i.e. the posterior cingulate cortex |

PCA = principal component analysis; SP = senile plaques; NFT = neurofibrillary tangles; AD = Alzheimer’s disease; CAA = cerebral amyloid angiopathy; MRI = magnetic resonance imaging; CA1 = cornu Ammonis subfield 1 of the hippocampus; PiB-PET = Pittsburgh compound B – positron emission tomography; MCI = mild cognitive impairment; SDAT = senile dementia of the Alzheimer’s type.

**Supplementary Table 5. Frequency of the hippocampal-sparing AD subtype – comparison of findings when using hippocampus versus entorhinal cortex for subtyping**

|  | **New data using the ADNI cohort** | | | | | |
| --- | --- | --- | --- | --- | --- | --- |
| Study | Mohanty  et al., 2020 ^8^  (Byun’s ^15^ algorithm - hippocampus) | Mohanty  et al., 2020 ^8^  (Byun’s ^15^ algorithm – entorhinal cortex) | Mohanty  et al., 2020 ^8^  (Charil’s ^6^ algorithm - hippocampus) | Mohanty  et al., 2020 ^8^  (Charil’s ^6^ algorithm – entorhinal cortex) | Mohanty  et al., 2020 ^8^  (Risacher’s ^20^ algorithm - hippocampus) | Mohanty  et al., 2020 ^8^  (Risacher’s ^20^ algorithm – entorhinal cortex) |
| Data modality | PET  (flortaucipir) | PET  (flortaucipir) | PET  (flortaucipir) | PET  (flortaucipir) | PET  (flortaucipir) | PET  (flortaucipir) |
| Braak’s tau NFT stage | unknown | unknown | unknown | unknown | unknown | unknown |
| Subtyping algorithm | Byun | Byun | Murray | Murray | Murray | Murray |
| Sample size | 84 | 84 | 84 | 84 | 84 | 84 |
| Percentage of individuals with hippocampal-sparing AD | 21% | 18% | 10% | 8% | 11% | 8% |
| Percentage of individuals with NFT count or tau PET uptake completely sparing the hippocampus, according to: |  |  |  |  |  |  |
| Neuropathologic definition | - | - | - | - | - | - |
| *‘Accuracy-based cut point’* | 0% | 0% | 0% | 0% | 0% | 0% |
| *‘+1SD cut point’* | 21% | 18% | 6% | 7% | 7% | 7% |
| *‘10% cut point’* | 21% | 18% | 6% | 7% | 7% | 7% |
| *‘Schöll cut point’* | 0% | 0% | 0% | 0% | 0% | 0% |
| *‘Maass cut point’* | 0% | 0% | 0% | 0% | 0% | 0% |

AD = Alzheimer’s disease; ADNI = Alzheimer's Disease Neuroimaging Initiative; NFT = neurofibrillary tangles; SD = standard deviation; pc = percentile; PET = positron emission tomography.
